# A phase I/II multicentric trial of gemcitabine and epirubicin in patients with advanced pancreatic carcinoma

**DOI:** 10.1038/sj.bjc.6603174

**Published:** 2006-05-23

**Authors:** A Eickhoff, W Martin, D Hartmann, J C Eickhoff, M Möhler, P R Galle, J F Riemann, R Jakobs

**Affiliations:** 1 Medical Department C, Klinikum Ludwigshafen gGmbH, Bremserstr.79, Ludwigshafen D-67063, Germany; 2 Department of Biostatistics and Medical Informatics, University of Wisconsin, Madison, WI, USA; 3 I. Medical Clinic, Johannes Gutenberg University Mainz, Mainz, Germany

**Keywords:** pancreatic cancer, chemotherapy, gemcitabine, epirubicin

## Abstract

Potential synergistic interaction between gemcitabine (GEM) and epirubicin (EPI) in pancreatic cancer have been described previously. The maximum-tolerated dose in this trial was GEM 1000 mg m^−2^ and EPI 45 mg m^−2^. Median time to progression was 5.1 months and median survival time 7.4 months. This combination appears well tolerated and shows promising clinical activity.

Pancreatic cancer is one of the most aggressive human cancers with an extremely poor prognosis. In 1998, gemcitabine (GEM) was approved for use in the palliative treatment approach (Burris *et al*, 1997). However, even with GEM, monotherapy has obvious limitations in advanced pancreatic cancer and various combinations with other agents have been investigated in phase III trials as well (Burris, 2005).

*In vitro* studies in solid tumour cell lines have demonstrated that pretreatment of cancer cells with the anthracycline epirubicin (EPI), followed by administration of GEM, inhibits proliferation and increases the rate of DNA fragmentation (Angelucci *et al*, 1997). The aim of this study was to find the maximum-tolerated dose (MTD) of EPI in combination with GEM in pancreatic carcinoma. We also sought to evaluate the antitumour activity and the clinical benefit of this combination.

## MATERIALS AND METHODS

### Patient eligibility

Patients with unresectable pancreatic carcinoma with or without distant metastases were eligible for the trial. Eligibility criteria included age 18–75 years, Karnofsky's performance status (KPS) ⩾60 and a left ventricular ejection fraction measured by multiple electrocardiogram-gated radionuclide study (MUGA-scan) higher than 50%. Baseline laboratory requirements were defined as described elsewhere (Hess *et al*, 2003). The study was carried out in accordance with the Declaration of Helsinki and approved by the local ethics committees; written informed consent was obtained from all patients.

### Treatment

Gemcitabine (Gemzar®, Eli Lilly, Indianapolis, IN, USA) was administered as a 30-min i.v. infusion on days 1 and 8; EPI (Pharmacia, Erlangen, Germany) as an i.v. bolus injection over a period of 5 min prior to GEM on day 8 of each 21-day cycle. In phase I the dose escalation to a maximum of six levels consisted of: Step 1: GEM 800 mg m^−2^, EPI 35 mg m^−2^ until Step 6: GEM 1000 mg m^−2^, EPI 90 mg m^−2^.

### Toxicity score

Dose-limiting toxicity was defined, using the World Health Organization (WHO) criteria (Miller *et al*, 1981). Complete blood cell counts were measured at days 1, 8 and 15 of each 21-day cycle.

### Maximum-tolerated dose for phase I of gemcitabine and epirubicin

An open-label, phase I trial design with a standard dose escalation schedule was used to determine the MTD. The MTD was defined as the highest safely tolerated dose where at most one patient experienced dose-limiting toxicity (DLT) with the next higher dose having at least two patients who experienced DLT. A DLT was defined as described elsewhere (Van Putten *et al*, 2000). Patients enrolled in cohorts of three. If no patients in a cohort experienced DLT in the first two cycles, escalation proceeded to the next higher dose level. If one of the first three patients experienced a DLT, three additional patients were added in that cohort. If no additional DLT's occurred, escalation continued. The MTD represents the dose recommended for the phase II trial.

### Assessment of safety, efficacy and quality of life

Adverse events reported according to the standard World Health Organization (WHO) criteria were used for safety assessment (Miller *et al*, 1981). Evaluation of tumour response was conducted according to standard WHO criteria. A responder was defined as any patient who had a complete or partial response, which was confirmed by a second evaluation with the same imaging technique at least 4 weeks later. Quality of life was measured with a standardised and validated questionnaire (Spitzer Quality of Life Index) and clinical benefit response was defined as in the study by Burris *et al* (1997).

### Statistical analysis

Toxicity and efficacy were summarised by descriptive statistics. The tumour response was presented as percentage along with the 95% confidence interval (CI). The significance level for each hypothesis test was 5%. Median time to progression and overall survival time was calculated according to the Kaplan–Meier product-limit method. Subgroups were compared using the log-rank test.

## RESULTS

Between May 2000 and 2003, a total of 40 symptomatic patients were enrolled in the study (Table 1). A total of 230 cycles of chemotherapy was administered with a median of six cycles per patient (range 2–9).

Three patients were entered at each dose level 1, 2 and 3 in the phase I part. Six patients were assigned to dose level 4. At that dose level, two of six patients experienced DLT, which consisted of grade 3 neutropenia and anaemia in one patient and a febrile grade 4 neutro- and thrombocytopenia in a second patient. Therefore, dose level 3 (GEM 1000 mg m^−2^ and EPI 45 mg m^−2^), was found to be the MTD for the phase II part. Overall, 28 patients were treated with GEM 1000 mg m^−2^ and EPI 45 mg m^−2^.

### Toxicity

Haematological toxicity observed in the phase I part is shown in Table 2. Nonhaematological toxicity in these patients was mainly mild nausea/vomiting (grade 1/2). Two patients had grade 3 alopecia and mild gastrointestinal toxicity (diarrhoea) occurred in four patients. Five patients (33%) experienced grades 1 and 2 mucositis, but this was manageable in all patients. A dose reduction was necessary in four patients, three of whom were treated at dose level 4. Renal, hepatic and neurologic toxicities were generally mild and no evidence of cardiac toxicity was recorded.

Overall, the 28 patients who were treated with the MTD of GEM 1000 mg m^−2^ and EPI 45 mg m^−2^ experienced only mild toxicity. Grade 3 haematologic toxicity occurred in one patient of phase I (anaemia) and one patient of phase II (neutropenia). In seven (25%) patients dose reduction of both drugs to 75% was necessary while one patient received dose reduction to 50% due to leukopenia grade III in one cycle, and treatment delay for 1 week in another cycle.

One patient at this dose level experienced grade 3 nausea/vomiting. Chemically induced phlebitis due to EPI was noted only in one other patient. Hospitalisation was necessary for another patient due to pneumonia. No major hepatic, renal, cardiac and neurologic toxicities were seen as well (Table 3).

### Tumour response and survival

The overall response rate was 23% (95% CI: 0.29–0.62) based on nine of 40 patients with partial response. The median time to response was 3 (range 2.5–3.7) months, and the median duration of response was 8.2 (range 4–12) months. Stable disease (SD) occurred in 14 of 40 (35%) patients. The remaining 17 (42.5%) patients showed progressive disease. Median time to disease progression for all patients was 4.2 (range 2–12) months.

The 6 months and 1-year survival rates were 55% (22/40 patients) and 22.5% (9/40 patients), respectively. The median overall survival time was 7.4 (range 2–21.8) (95% CI: 5.1–9.9) months. For patients with tumour response (9/40 patients), the median survival was 12.6 (range 10.7–21.8) months, and the probability of surviving beyond 1 year was 55%. Patients with objective tumour response survived significantly longer than patients with no tumour response (*P*=0.01).

### Clinical benefit response

Thirty-seven patients with tumour-related symptoms were considered evaluable for clinical benefit response. In 10/35 (29%) patients suffering from pain at study entry, pain intensity and/or analgesic use was reduced at least by 50% compared to baseline values, and 18 remained stable in this category. Improvement of KPS occurred in 13 (35%) patients and performance status remained stable for 20 (54%) patients during at least 4 weeks. Eight patients (21.6%) had a weight gain of ⩾7% from baseline during the study. Improvement of the quality of life during treatment measured and expressed with the Spitzer-activity-index was seen in 15 of 37 (40.5%) patients. In the final assessment, a total of 18 patients were classified as clinical benefit responders, yielding a clinical response rate of 49% (95% CI: 0.29–0.62). We observed a significant correlation between clinical benefit response and objective tumour response as well (*P*<0.001).

## DISCUSSION

Our experience with GEM and EPI clearly demonstrated that this combination is adequately tolerated with moderate toxicities that were qualitatively and quantitatively similar to those reported from other trials (Scheithauer *et al*, 1999; Neri *et al*, 2002; Reni *et al*, 2005).

In line with the preclinical observation of synergy, the combination of GEM and EPI achieved a promising preliminary antitumour effect. In this trial, we achieved a 22.5% overall remission rate in 40 evaluable patients and a median response duration of 8 months. With an additional 35% of patients experiencing SD this combination achieved a favourable disease control in almost 60% of patients. Three other GEM+EPI studies in pancreatic cancer have been published in manuscript form (Scheithauer *et al*, 1999; Neri *et al*, 2002; Reni *et al*, 2005).

Concerning objective tumour response and disease control, the reported data are comparable (objective response 21–51%; disease control 65–80%) with our results. Neri *et al* (2002) reported an overall response rate of 25% and a SD in 41% of their patients. Most patients (77%) in that study were stage IV and two-thirds had a mean KPS between 70 and 80.

Comparable objective results were published by Scheithauer *et al* (1999) with a combination of GEM 1000 mg m^−2^+EPI 60 mg m^−2^+granulocyte colony-stimulating factor. Recently, a phase III trial with GEM in combination with EPI, 5-FU and cisplatin *vs* GEM was published by Reni *et al* (2005). They found an overall response of 38.5% and stabilisation of the disease in further 28.8% with the combination therapy. Overall, these data from GEM+EPI trials supports the hope that some improvements in response rates and prognosis may be achievable.

In our study, we observed a clinical benefit response in 49% of symptomatic patients. This compares favourably with the rate of clinical benefit response in the GEM+EPI studies by Scheithauer *et al* (1999) (43%) and Neri *et al* (2002) (44%). However, using the same validated definitions for clinical benefit response, there is almost a doubling of the rate of clinical benefit responders with the combination therapy in comparison with GEM alone (Burris *et al*, 1997, Neri *et al*, 2002).

In conclusion, this combination chemotherapy of GEM and EPI was well tolerated and feasible at the recommended dose level. The regimen is active with a high rate of clinical benefit responders. Whether addition of EPI to GEM is comparable to the combination of other drugs (cisplatin, irinotecan, 5-FU, and ‘biologicals’) and GEM should be further evaluated in randomised phase III trials.

## Figures and Tables

**Table 1:** Pretreatment characteristics

**Characteristics**	***N* (%)**
Number of patients	40
Age, median/range (years)	67.5±8.5 (43–75)
Gender (F/M)	16/24 (40/60)

*Karnofsky performance status (KPS)*	
⩾60	8 (20)
70–80	25 (62.5)
90–100	7 (17.5)

*Disease stage at presentation*	
Stage III	13 (32)
Stage IV	27 (68)

*Histological grade*	
G 1	3 (7.5)
G 2	26 (65)
G 3	11 (27.5)

*Prior surgery*	
None	24 (60)
Explorative laparotomy	10 (25)
Palliative bypass	6 (15)

**Table 2:** Haematological toxicity (number of patients) in the phase I part of the study

**WHO toxicity**	**Leucocytes**	**Granulocytes**	**Haemoglobin**	**Platelets**
*Level 1*				
0–1	0	2	3	3
2	3	1	0	0
3	0	0	0	0
4	0	0	0	0

*Level 2*				
0–1	2	1	2	3
2	1	2	1	0
3	0	0	0	0
4	0	0	0	0

*Level 3*				
0–1	1	2	1	3
2	2	1	1	0
3	0	0	1	0
4	0	0	0	0

*Level 4*				
0–1	4	2	3	3
2	0	2	2	2
3	1	1	1	0
4	1	1	0	1

Level 1: GEM 800 mg m^−2^+EPI 35 mg m^−2^; Level 2: GEM 800 mg m ^2^+EPI 45 mg m^−2^; Level 3: GEM 1000 mg m^−2^+EPI 45 mg m^−2^; Level 4: GEM 1000 mg m^−2^+EPI 60 mg m^−2^.

**Table 3:** Summary of maximum treatment-associated toxicities (*n*=25) in the phase II part of the study

	**Number of patients/WHO-toxicity grade (%)**			
**Toxicity**	**1**	**2**	**3**	**4**
*Haematological and other laboratory-based toxicity*				
Leukopenia	3 (12)	4 (16)	1 (4)	0
Granulocytopenia	2 (8)	3 (12)	1 (4)	0
Thrombocytopenia	0	0	0	0
Anaemia	3 (12)	1 (4)	0	0
Bilirubin	1 (4)	0	0	0
Alkaline phosphatase	3 (12)	1 (4)	0	0
Serum transaminases	3 (12)	0	0	0

*Symptomatic toxicity*				
Nausea/vomiting	4 (16)	1 (4)	1 (4)	0
Stomatitis	2 (8)	0	0	0
Diarrhoea	2 (8)	0	0	0
Constipation	1 (4)	0	0	0
Infection	1 (4)	0	1 (4)	0
Fever	2 (8)	0	0	0
Alopecia	4 (16)	1 (4)	1 (4)	0
Cutaneous	0	0	0	0
Phlebitis	1 (4)	1 (4)	0	0
